# The influence of environmental temperature on appetite-related hormonal responses

**DOI:** 10.1186/s40101-015-0059-1

**Published:** 2015-05-03

**Authors:** Chihiro Kojima, Hiroto Sasaki, Yoshifumi Tsuchiya, Kazushige Goto

**Affiliations:** Graduate School of Sport and Health Science, Ritsumeikan University, Kusatsu, Shiga Japan; Faculty of Sport and Health Science, Ritsumeikan University, 1-1-1, Nojihigashi, Kusatsu, Shiga 525-8577 Japan

**Keywords:** Appetite regulation, Ghrelin, PYY, Environmental temperature

## Abstract

**Background:**

Acute exercise in the heat has been shown to reduce appetite. However, the influence of exercise in the cold on appetite regulation remains unclear. The aim of this study was to compare exercise-induced appetite regulation under three different environmental temperatures.

**Methods:**

Eleven male participants completed three experimental trials on the following separate days: exercise in the heat (36°C), exercise at neutral temperature (24°C), and exercise in the cold (12°C). The exercise trials consisted of pedaling exercises for 30 min at 65% of maximal oxygen uptake (VO_2max_). Blood samples were collected repeatedly to determine plasma ghrelin, peptide YY (PYY) and other hormonal concentrations. Subjective feelings of hunger and tympanic temperature were also monitored.

**Results:**

Tympanic temperature was significantly higher in the 36°C trial than that of the other two trials (*P* < 0.05). The subjective feelings of hunger in the 36°C and 24°C trials were significantly lower than those in the 12°C trial (*P* < 0.05). Plasma ghrelin concentration decreased significantly with exercise in all conditions (*P* < 0.05), and the responses were not significantly different among the three conditions. Plasma PYY concentration increased significantly after the exercise in the 24°C trial only (*P* < 0.05), with no significant difference among the three trials.

**Conclusions:**

These results suggest that exposure to hot or cold temperatures during exercise did not affect exercise-induced plasma ghrelin and PYY responses. However, the exercise-induced reduction of subjective hunger was significantly attenuated in a cold environment.

## Background

Appetite regulation has a complex mechanism that is closely related to the levels of several circulating hormones. For example, plasma ghrelin secreted from the stomach promotes strong hunger and food intake [1]. In contrast, multiple hormones secreted from the gastrointestinal tract have an anorexigenic effect. Peptide YY (PYY) and glucagon-like peptide (GLP-1) play a role in attenuating appetite [2-5].

In recent years, exercise has been shown to affect acute appetite-regulating hormones [6-11]. King *et al*. [9] showed that running on a treadmill for 90 min caused a significant reduction in plasma ghrelin concentration and in the subjective feeling of hunger. Martins *et al*. [6] reported that GLP-1 and PYY concentrations decreased significantly after endurance exercise at 65% of maximal oxygen uptake (VO_2max_). Furthermore, energy intake during a buffet-style meal decreased significantly after exercise compared with that of the control condition without exercise. Moreover, endurance exercise at higher intensity (75% of VO_2max_) caused a significantly greater elevation in plasma GLP-1 and PYY concentrations and subsequent attenuation of energy intake compared with endurance exercise at lower intensity (50% of VO_2max_) [8]. These findings suggest that endurance exercise impairs appetite and energy intake by modulating orexigenic and anorexigenic hormonal secretions.

Exercise-induced appetite-regulating hormonal responses are influenced by environmental conditions. Among several environmental factors during exercise, temperature appears to be the most practical and easy to manipulate. Tomasik *et al*. [12] demonstrated that plasma ghrelin concentration was significantly higher at 2°C than that at 30°C. At least two previous studies have focused on the influence of environmental temperature during exercise on subsequent energy intake. Shorten *et al*. [13] compared appetite regulation among three different conditions, consisting of exercise at 36°C, exercise at 25°C, and rest at 25°C. Exercise at 36°C tended to reduce the plasma ghrelin concentration compared with the other two conditions. Furthermore, energy intake during a buffet-type meal 30 min after exercise was significantly lower in the exercise condition at 36°C than the rest condition at 25°C. Wasse *et al*. [14] compared changes in appetite regulation under different environmental temperatures in two separate experiments (exercise at 30°C *versus* 20°C for experiment 1 and exercise at 20°C *versus* 10°C for experiment 2). Although no significant differences in plasma ghrelin concentrations were observed between conditions in either study, energy intake was lower after exercise at 30°C (experiment 1) and higher after exercise at 10°C (experiment 2). However, the above results may have been influenced by individual differences because different subjects were recruited for the two experiments. No study has compared appetite regulation with exercise in cold, neutral, and hot environments in the same subjects. Therefore, the aim of this study was to compare appetite-regulating hormonal responses after exercise in hot, neutral, and cold environments. We hypothesized that appetite would be attenuated with an increase in environmental temperature during and after exercise.

## Methods

### Subjects

Eleven male subjects [mean (± standard error) age, 21.1 ± 0.5 years; height, 173.7 ± 2.7 cm; weight, 66.1 ± 4.0 kg; body mass index, 21.7 ± 2.7 kg/m^2^] participated in this study. The subjects were informed about the purpose, experimental procedure, and risk of the study, and written informed consent was obtained from all the participants. This study was approved by the Ethics Committee for Human Experiments at the Ritsumeikan University, Japan.

### Experimental design

All subjects completed three sessions on different days. Each session was separated by at least 1 week. The subjects conducted three experimental trials in a randomized counterbalanced design as follows: a trial with exercise in the heat (36°C), a trial with exercise at a normal temperature (24°C), and a trial with exercise at a cold temperature (12°C). Exercise-induced metabolic and hormonal responses and subjective appetite regulation during and after exercise were compared among the three trials.

### Exercise trials

The exercise trials consisted of 30 min of pedaling exercise on a cycling ergometer (Aerobike75XL III, Konami Sports Life, Tokyo, Japan) at 65% of VO_2max_. Pedaling frequency was set to 70 rpm. All sessions were performed in an environmentally controlled chamber. The environmental temperature was set to 36°C, 24°C, or 12°C for each condition. Relative humidity was maintained at 40%. Subjects ingested 100 ml of water at 10°C, 20°C, and 30 min during exercise under each condition. The subjects remained in the chamber for 30 min after exercise to evaluate metabolic and hormonal responses.

### Measurements

#### *VO*_*2max*_

VO_2max_ was assessed at a normal temperature (24°C) by an incremental pedaling test using a cycling ergometer to determine workload during the subsequent three experimental trials. The test began at 70 W, and load progressively increased by 30 W until voluntary exhaustion. The test was terminated when the subject failed to maintain the prescribed pedaling frequency of 70 rpm or reached the VO_2_ plateau. Respiratory gasses were collected during exercise and analyzed using an automatic gas analyzer (AE310S, Minato Medical Science Co., Ltd., Tokyo, Japan) to evaluate VO_2_, carbon dioxide output, and ventilatory volume. Data were averaged every 30 s.

### Blood sampling

Subjects arrived at the laboratory in the morning of the experimental trial days, following an overnight fast (at least 12 h). A polyethylene catheter was inserted into an antecubital vein after a 20 min rest, and a baseline blood sample was obtained. A series of blood samples was collected at 15 min and 30 min during exercise and 30 min after exercise. Serum and plasma samples were obtained after a 10 min centrifugation at 4°C, and the serum and plasma samples were stored at −80°C until analysis. Plasma ghrelin, serum growth hormone (GH), insulin, free fatty acid (FFA), glycerol, blood glucose, and lactate concentrations were measured. Blood glucose and lactate concentrations were measured using a glucose analyzer (Free style, Nipro Co., Osaka, Japan) and a lactate analyzer (Lactate Pro, Arkray Co., Kyoto, Japan) immediately after blood collection, respectively. Blood glucose concentrations were measured in duplicate, and average values were used for analysis. Serum GH, insulin, and FFA concentrations were measured in a clinical laboratory (SRL Inc., Tokyo, Japan). The intra-assay coefficient of variation (CV) was 2.1%, 3.1%, and 2.6%, respectively. Plasma ghrelin concentrations were measured using an enzyme-linked immunosorbent assay (ELISA) kit (Mitsubishi Chemical Medicine Corp., Tokyo, Japan). The intra-assay CV was 4.8%. Plasma PYY concentrations were measured by an ELISA kit (Phoenix Pharmaceuticals, Inc., USA). The intra-assay CV was 8.9 %. Serum glycerol was measured with a commercially available kit from Caymen Chemical Co. (Ann Arbor, MI, USA). The intra-assay CV was 1.9%.

### Subjective feelings of hunger, motivation to eat, and satiety

Ratings of subjective feelings of hunger, motivation to eat, and satiety were evaluated using a 100-mm visual analog scale [15] before exercise, at 15 min and 30 min during exercise and 15 min and 30 min after exercise.

### Tympanic temperature

Tympanic temperature was evaluated using an infrared thermometer before exercise at 15 min and 30 min during exercise and 15 min and 30 min after exercise [13]. Measurements at each time point were repeated three times, and average values were used.

### Statistical analyses

Data are expressed as means ± standard errors. Time-course changes in the blood parameters, tympanic temperature, and subjective appetite were compared using a two-way repeated-measures analysis of variance (ANOVA) to determine the interaction (trial × time) and main effects (trial and time). When the ANOVA revealed a significant interaction or main effect, the Tukey–Kramer test was used. Statistical significance was accepted at a *P* value <0.05.

## Results

### Tympanic temperature

**Figure 1:**
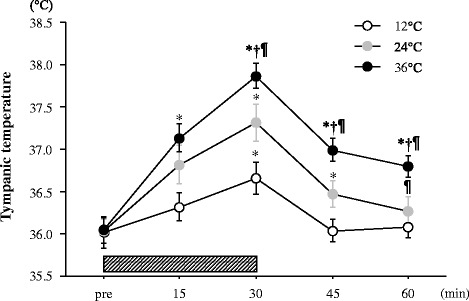
Change in tympanic temperature. The *shaded bar* indicates the duration of exercise. Values are means ± SE. †; Significant difference *versus* 24°C. ¶; Significant difference *versus* 12°C.*; Significant difference *versus* Pre.

Figure 1 shows the changes in tympanic temperature during each trial. A significant interaction (trial × time) and main effect for trial and time were observed (*P* < 0.05). All the trials showed significant increases in tympanic temperature during exercise, and temperature remained elevated after exercise in the 36°C and 24°C trials (*P* < 0.05). Tympanic temperature was significantly higher during and after exercise in the 36°C trial than that of the other two trials (*P* < 0.05). The 24°C trial showed significantly higher values compared with that of the 12°C trial after the exercise period (*P* < 0.05).

### Blood parameters

Table 1 shows the changes in blood lactate and glucose and serum FFA and glycerol concentrations. No significant differences were observed among the three trials for any of the blood variables at baseline (before exercise). Blood lactate concentration increased significantly in all the trials (*P* < 0.05). The 36°C trial showed significantly higher values during the exercise and post-exercise periods, compared with values in the 24°C and 12°C trials, respectively (*P* < 0.05). Blood glucose concentrations decreased significantly with exercise in all the trials (*P* < 0.05), and no significant differences were observed among the three trials at any time point. Similarly, FFA concentrations decreased significantly during exercise in all the trials (*P* < 0.05), and no significant difference was observed among the three trials at any time point. Serum glycerol concentrations increased significantly in all the trials (*P* < 0.05). Serum glycerol concentration was significantly higher in the 36°C trial at 30 min during exercise than that of the 24°C and 12°C trials, respectively (*P* < 0.05).

**Table 1:** **Changes in blood parameters**

		**Pre**	**15 min**	**30 min**	**60 min**
Lactate	12°C	1.3 ± 0.1	6.1 ± 0.6*	4.8 ± 0.4*	2.7 ± 0.3
(mmol/L)	24°C	1.1 ± 0.1	4.9 ± 0.3*¶	4.8 ± 0.3*	2.0 ± 0.2
	36°C	1.2 ± 0.1	5.3 ± 0.2*	5.5 ± 0.3†¶*	2.1 ± 0.2†¶
Glucose	12°C	94 ± 2	80 ± 3*	80 ± 3*	85 ± 2*
(mg/dL)	24°C	92 ± 2	78 ± 2*	81 ± 2*	86 ± 2*
	36°C	92 ± 2	81 ± 2*	85 ± 3	91 ± 2
FFA	12°C	393 ± 44	285 ± 32*	308 ± 46*	462 ± 58
(μEq/L)	24°C	456 ± 95	211 ± 30*	272 ± 38*	431 ± 74
	36°C	477 ± 93	227 ± 28*	312 ± 46*	582 ± 46*
Glycerol	12°C	2.5 ± 0.9	5.8 ± 2.1	5.3 ± 1.2*	7.1 ± 2.9*
(mg/dL)	24°C	3.2 ± 0.8	4.6 ± 1.8	5.2 ± 1.4*	2.8 ± 0.8*
	36°C	3.1 ± 0.6	6.9 ± 2.6*	7.6 ± 1.7†¶*	4.6 ± 1.4*

Values are means ± SE. †; Significant difference *versus* 24°C. ¶; Significant difference *versus* 12°C. *; Significant difference *versus* Pre.

**Figure 2:**
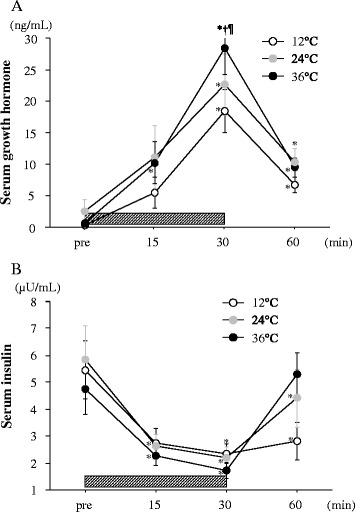
Change in serum growth hormone **(A)** and serum insulin **(B)** concentrations. The *shaded bar* indicates the duration of exercise. Values are means ± SE. †; Significant difference *versus* 24°C. ¶; Significant difference *versus* 12°C. *; Significant difference *versus* Pre.

Figure 2 shows the changes in serum GH and insulin concentrations. Serum GH concentrations increased significantly with exercise in all the trials (*P* < 0.05). However, the magnitude of the exercise-induced GH response was significantly greater in the 36°C trial compared with the other two trials (*P* < 0.05). Serum insulin concentrations decreased significantly with exercise in all the trials (*P* < 0.05). However, no significant difference was observed among the three trials at any time point.

**Figure 3:**
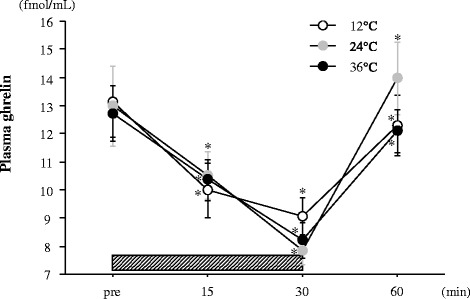
Change in plasma ghrelin concentrations. The *shaded bar* indicates the duration of exercise. Values are means ± SE. *; Significant difference *versus* Pre.

Figure 3 shows the changes in plasma ghrelin concentrations. No significant interaction (trial × time) or main effect for trial was observed. Exercise in all the trials significantly reduced plasma ghrelin concentration (*P* < 0.05). However, no significant difference in plasma ghrelin concentration was observed among the three trials.

**Figure 4:**
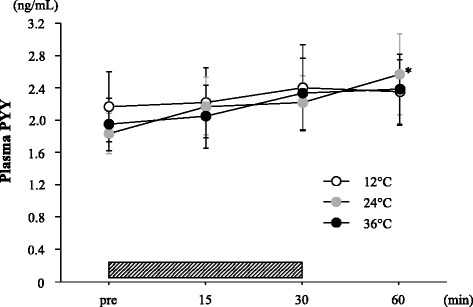
Change in plasma PYY concentration. The *shaded bar* indicates the duration of exercise. Values are means ± SE. *; Significant difference *versus* Pre.

Figure 4 shows the changes in plasma PYY concentrations. Plasma PYY concentrations in the 24°C was elevated significantly after exercise (*P* < 0.05). However, no significant difference was observed among three trials at any point.

### Subjective feeling of appetite

Table 2 shows the time-course changes in the subjective feeling of appetite. Hunger decreased significantly with exercise in all the trials (*P* < 0.05). Moreover, exercise-induced hunger attenuation was more apparent in the 36°C and 24°C trials than that of the 12°C trial (*P* < 0.05). These differences remained evident during the post-exercise period (*P* < 0.05). No significant difference in hunger was observed between the 24°C and 36°C trials at any time point (*P* < 0.05). The motivation to eat decreased significantly with exercise in all the trials (*P* < 0.05). The exercise-induced attenuation of the motivation to eat was significantly greater in the 36°C and 24°C trials than that of the 12°C trial during the exercise and post-exercise periods (*P* < 0.05). Satiety was significantly elevated with exercise in the 12°C and 36°C trials (*P* < 0.05). However, satiety was not significantly different among the trials at any time point.

**Table 2:** **Changes in subjective feeling of appetite**

		**Pre**	**15 min**	**30 min**	**45 min**	**60 min**
Hunger (mm)	12°C	73 ± 3	56 ± 7*	43 ± 9*	67 ± 5	74 ± 5
	24°C	57 ± 6¶	38 ± 6*¶	24 ± 5*¶	45 ± 6¶	58 ± 5
	36°C	69 ± 6	38 ± 9*¶	34 ± 8*	45 ± 9*¶	53 ± 1¶
Motivation to eat (mm)	12°C	65 ± 6	46 ± 7*	35 ± 8*	68 ± 6	69 ± 8
	24°C	48 ± 7¶	24 ± 6*¶	21 ± 6*	37 ± 6¶	57 ± 3
	36°C	54 ± 8	30 ± 9*	28 ± 7*	49 ± 9¶	56 ± 1
Satiety (mm)	12°C	15 ± 3	32 ± 1	33 ± 1*	20 ± 6	14 ± 4
	24°C	23 ± 5	33 ± 7	28 ± 6	31 ± 7	16 ± 4
	36°C	24 ± 7	36 ± 9*	35 ± 1*	31 ± 9	21 ± 9

Values are means ± SE. ¶; Significant difference *versus* 12°C. *; Significant difference *versus* Pre.

## Discussion

The present study was designed to determine the influence of environmental temperature during exercise on appetite regulation. The main finding was that exposures to a hot (36°C) or cool (12°C) environment during exercise and post-exercise did not affect the exercise-induced plasma ghrelin and PYY responses. However, exercise-induced suppression of the subjective feeling of hunger was attenuated when exercise was conducted in a cool environment.

Tympanic temperature increased significantly during exercise. Rectal or esophageal temperature is generally used as an indicator of core temperature during exercise. We selected tympanic temperature in accordance with a previous study that determined the influence of environmental temperature on exercise-induced appetite regulation [13]. Consequently, tympanic temperature during and after exercise was significantly higher in the 36°C trial than that of the 24°C and 12°C trials. The 24°C trial also showed significantly higher values compared with those of the 12°C trial. Therefore, the difference in environmental temperature during each trial affected body temperature.

In accordance with previous studies [8,9,16,17], exercise significantly decreased the subjective feeling of hunger in all the trials. However, the magnitude of the exercise-induced suppression of hunger was significantly impaired when exercise was performed under a cool condition (12°C). Exercise intensity is thought to be a key factor for reducing subjective hunger [8]. Additionally, environmental temperature has been suggested to affect appetite regulation. Wasse *et al*. [18] found that subjective hunger and energy intake were lower after exercise in a hot environment (30°C) than those after exercise in a cool environment (10°C). The present findings of a smaller exercise-induced reduction in hunger at 12°C may partially support this result. The mechanism of altered appetite regulation at different temperatures is complex, but thermal status may be involved in feeding responses [18]. Gastric emptying may also influence ingestive behavior and energy intake during subsequent meals [18]. Stengel *et al*. [19] showed that augmenting the gastric emptying response following cold exposure is associated with elevated acylated ghrelin concentration, which promotes energy intake. Several gut hormones - including PYY, GLP-1 and pancreatic polypeptide - play important roles in gastric function and appetite regulation [18]. In the present study, the plasma ghrelin concentration decreased significantly within 30 min of sub-maximal exercise in all the trials. However, no significant interaction or main effect for trial was observed for the plasma ghrelin response. Tomasik *et al*. [12] showed that ghrelin concentration increased after cold exposure compared with after neutral and hot exposure. However, information regarding the influence of different environmental temperatures on exercise-induced appetite-related hormonal (for example, ghrelin and PYY) responses is limited; only three studies have been published [13,14,20]. Shorten *et al*. [13] reported no difference in exercise-induced ghrelin responses at different temperatures, despite the fact that the relative energy intake after exercise was lower under a hot condition than under the control condition without exercise. Wasse *et al*. [14] reported that energy intake tended to be lower under a hot condition than under a neutral temperature condition (*P* = 0.08), whereas energy intake was higher under a cool condition than under a neutral condition (*P* = 0.08). However, ghrelin concentrations were not related to these differences in energy intake. Crabtree *et al*. [20] reported that ghrelin concentrations and energy intake after exercise in a cold environment were higher compared with those in a neutral temperature. These findings suggest that exposure to the cold rather than exposure to hot or neutral conditions promotes energy intake, but the association between this phenomenon and ghrelin response is inconclusive. We also determined the exercise-induced plasma PYY response in the present study, which was associated with impaired appetite. As reported in previous studies [8,21], plasma PYY concentration increased significantly after exercise in the 24°C trial only. However, a significant increase in plasma PYY concentration was not observed in the 12°C or 36°C trials. In addition, no significant difference in plasma PYY was observed among the three trials. Crabtree *et al*. [20] reported that environmental temperature does not influence the exercise-induced plasma PYY response. In contrast, Shorten *et al*. [13] reported that exercise in hot conditions increased PYY concentration at the onset of a subsequent meal. Among previous studies and the present study, influence of exercise on PYY response is inconsistent and still not conclusive. Taken together, our findings suggest that attenuation of the exercise-induced decrease in hunger in the 12°C trial did not correspond to ghrelin or PYY concentration; thus, another mechanism may be responsible for the response.

The limitation of the present study was the absence of energy-intake data. However, this was the first study to compare the influence of exercise-induced appetite regulation in hot, neutral, and cold temperatures in the same subjects. Future studies should investigate whether the attenuated exercise-induced reduction in hunger under a cold temperature affects energy intake during a subsequent meal. Recent studies suggest that environmental factors during exercise (for example, oxygen concentration and ambient temperature) may directly affect appetite regulation [12-14,18,20]. From a practical point of view, ambient temperature is easy to manipulate. Therefore, findings from these experiments would help establish an effective exercise prescription to prevent obesity and promote health.

## Conclusions

In conclusion, the exercise-induced plasma ghrelin and PYY responses did not differ in a cold (12°C) and hot (36°C) environment. However, exposure to a cold environment (12°C) during exercise attenuated the exercise-induced reduction in hunger. These results suggest that the influence of different environmental temperatures on exercise-induced appetite regulation is relatively minor.

## Abbreviations

PYY: peptide YY
GLP-1: glucagon-like peptide
VO_2max_: maximal oxygen uptake
GH: growth hormone
FFA: free fatty acid
